# It Is Not Always Sepsis: Fatal Tachypnea in a Newborn

**DOI:** 10.1155/2018/7858192

**Published:** 2018-02-21

**Authors:** Rachel Levene, Elza Pollak-Christian, Ashish Garg, Michael Keenaghan

**Affiliations:** ^1^Department of Pediatrics, SUNY Downstate Medical Center, Brooklyn, NY, USA; ^2^Department of Pediatrics, Neonatal-Perinatal Section, University of Oklahoma Health Sciences Center, Oklahoma City, OK, USA; ^3^Department of Pediatric Cardiology, University of Miami, Miami, FL, USA; ^4^Department of Pediatrics, Kings County Hospital Center, Brooklyn, NY, USA

## Abstract

Coarctation of the aorta (CoA) is a congenital cardiac malformation that is well understood. Despite being well characterized, CoA is a commonly missed congenital heart disease (CHD) during the newborn period. We report a full-term nine-day-old male who presented to the pediatric emergency department (ED) with isolated tachypnea. After an initial sepsis workup, subsequent investigations revealed critical CoA. Because the primary workup focused on sepsis, there was a significant delay in prostaglandin E1 (PGE_1_) initiation. This case illustrates the importance of early CoA recognition and timely initiation of PGE_1_ in newborns who present with suspected sepsis along with tachypnea.

## 1. Case

A previously healthy full-term nine-day-old male presented to the pediatric ED with complaints of tachypnea, poor feeding, and decreased urine output for two days. Review of systems was significant for a sick contact with an upper respiratory infection. Otherwise, there were no reported fevers, upper respiratory symptoms, vomiting, diarrhea, seizure-like activity, skin color changes, rash, or recent travel of the infant. Birth history was unremarkable; he was born at a gestational age of 38 weeks via normal spontaneous vaginal delivery. APGARs were 9 and 9 at 1 and 5 minutes of life, respectively. Birth weight was 2610 grams, and New York State newborn screening was negative. All maternal serologies were normal, as was prenatal sonogram. The nursery course was uncomplicated, and the patient passed the critical congenital heart disease (CCHD) screening at 29 hours of life.

**Figure 1:**
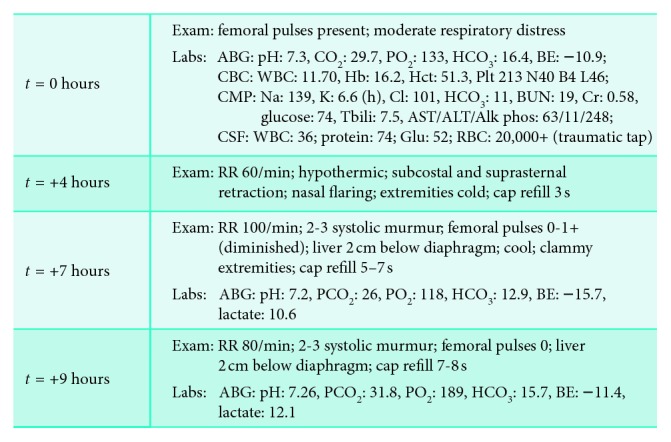
Disease progression of patient from ED (*t* = 0 hr) presentation to cardiogenic shock (*t* = +9 hr).

**Figure 2:**
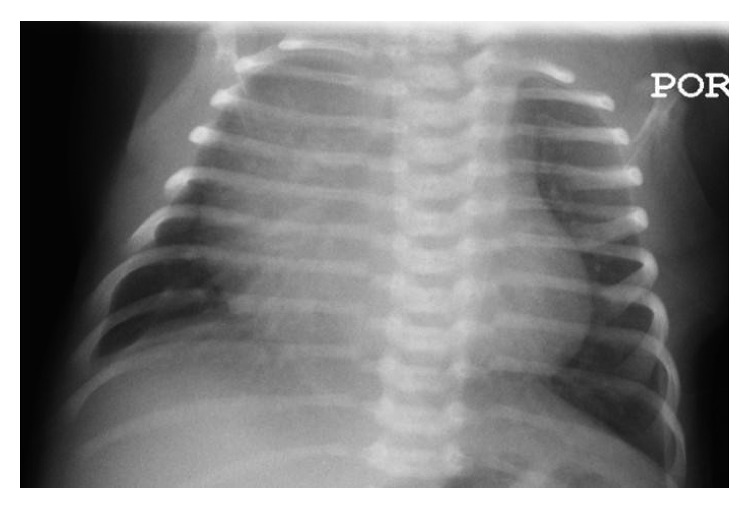
CXR (AP) shows a prominent thymus, normal cardiothoracic ratio, sharp costophrenic angles without cardiomegaly, consolidation, or increased perivascular markings.

Vital signs on presentation to the ED were temperature of 37.3°C, heart rate of 162 beats per minute, respiratory rate of 80 breaths per minute, blood pressure of 76/54 mmHg (right arm), and oxygen saturation of 95% on room air. The ED physical exam noted a newborn who appeared mildly dehydrated, with moderate distress as evidenced by retractions, tachypnea, and nasal flaring. There was no rhinorrhea and nasal congestion, and the lungs were clear to auscultation without any wheezing, rhonchi, or rales. Blood, urine, and CSF cultures were drawn, and ampicillin, cefotaxime and acyclovir were started. Nasopharyngeal viral swab was negative for respiratory viruses. CBC was normal for age, and CMP was significant for metabolic acidosis and hyperkalemia (Figure 1). Chest X-ray was unremarkable (Figure 2). The patient was then admitted to the pediatric intensive care unit (PICU) for worsening respiratory distress.

**Figure 3:**
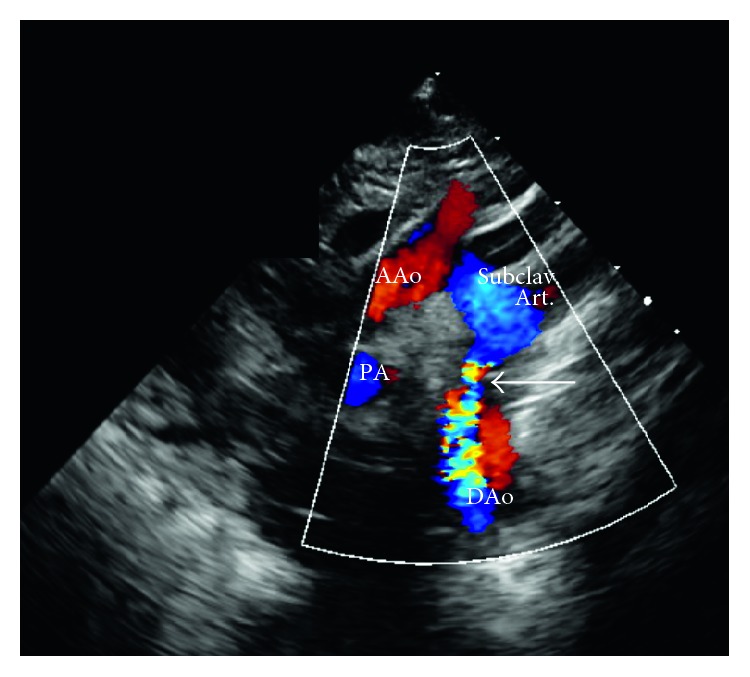
Color Doppler showing narrowing (arrow) with marked flow acceleration in descending aorta. AAo: ascending aorta; DAo: descending aorta.

In the PICU, patient was noted to have weak femoral pulses and no urine output after four hours from PICU admission. Vitals were significant for hypothermia (temperature of 34.8°C) and worsening tachypnea to 90 breaths per minute. Lower limb blood pressures at that time were undetectable (see Figure 2 for detailed exam). Alprostadil (PGE_1_) was started in the PICU. At initiation of PGE_1_, lactic acid level was 10.6 mg/dl. Critical coarctation of the aorta (CoA) was suspected, and 2D echocardiogram confirmed severe discrete coarctation of the aorta (Figure 3). After initial stabilization with prostaglandin and dopamine drips, patient received surgical correction on hospital day 4. Despite all interventions, the patient eventually succumbed to a human metapneumovirus infection on postoperative day 35.

## 2. Discussion

Despite constituting only 8–10% of congenital cardiac defects [1], CoA is the most missed congenital heart disease in the newborn period [2]. It is missed due to the presence of a patent ductus arteriosus (PDA) in the first 3 days of life, which maintains blood supply to the descending aorta from the pulmonary artery [3]. With majority of CoA lesions located proximal to the ductus arteriosus (DA), an infant will remain asymptomatic until the DA can no longer perfuse the descending aorta. Therefore, the extent of DA patency, along with the rapidity of its closure, determines the timing and severity of clinical presentation. Typically, this occurs after the first 48 hours of life. When the DA closes, blood supply to the lower extremities, kidneys, and other abdominal organs is compromised [4]. Thus, maintaining the patency of ductus arteriosus is vital to the survival of infants with critical CoA.

Nearly a quarter of CHD are defined as critical, meaning the defect requires surgery or catheter intervention within the first week of life [5]. With the recent implementation of CCHD screening, majority of infants with critical CHD are screened and identified prior to discharge from most nurseries. As of October 2016, 48 states have mandated CCHD pulse oximetry screening in the newborn nursery with legislation enacted or have initiated pilot programs [6]. CCHD screening is designed to detect asymptomatic newborns that have pulse oximetry readings of less than 95% after 24 hours of life [7]. Per the AAP algorithm, any pulse oximetry screening that is greater than or equal to 95% in both right lower and upper extremities or with less than or equal to 3% absolute difference in oxygen saturation between the right upper and lower extremity is considered a negative screen [7]. Despite the screen's sensitivity to detect approximately 76% of CCHD, CoA remains the most commonly missed CCHD lesion [8, 9]. A recent study reported that 75% of neonates with missed CCHD diagnoses had a form of aortic obstruction [10]. Though the implementation of CCHD screening was initiated in 2011, many physicians who work outside of neonatology are unfamiliar with the screening process and are unaware about which diagnoses can be missed through this screening process [11].

CoA should be considered in any neonate that presents with “silent tachypnea” defined as tachypnea without any other signs of upper respiratory infection such as rhinorrhea, nasal congestion, cough, or wheezing [4]. A history of concurrent poor feeding and decreased activity should also alert the physician to a diagnosis of CoA. Classically, in CoA, the physical exam will show upper extremity hypertension, decreased lower extremity blood pressures, diminished pulses in the lower extremities, hypothermia, and a cardiac murmur, though nearly 50% of infants do not present with a murmur [5]. Significant lactic acidosis is another clue to the diagnosis. Infants with CoA who develop congestive heart failure may also have oliguria or anuria, severe acidemia, circulatory shock, and differential cyanosis [4]. Therefore, clinicians should perform a careful palpation of pulses in any infant presenting in the neonatal period to the emergency department. The pulse discrepancy should be confirmed by blood pressure measurement in both arms and both legs.

Infants with critical cardiac lesions have an increased risk of morbidity and mortality when diagnosis and intervention is delayed [10]. While some neonates with critical CHD may present with obvious signs such as shock, others may appear asymptomatic or with subtle signs. This case underscores the importance of emergency physicians performing cardiovascular evaluations as part of the initial newborn physical assessment and to be mindful that the current methods of CCHD screening do not rule out the possibility of CoA or aortic obstructive lesions. Additionally, pediatric emergency room clinicians should be comfortable with initiating PGE_1_ in newborns with suspected sepsis and tachypnea until CHD is ruled out.

The management of CoA is twofold prior to corrective surgery, with efforts targeted at maintaining DA patency and controlling heart failure. In order to reopen and maintain DA patency, PGE_1_ infusion must be started immediately in order to reestablish adequate lower extremity blood flow. Dopamine or dobutamine should be started to improve contractility when concurrent heart failure is suspected. Supportive care to correct metabolic acidosis, hypoglycemia, respiratory failure, and anemia should be prioritized. It is important that clinicians are familiar with the side effects PGE_1_, including fever, hypotension, tachycardia, and dose-dependent apnea [12]. Due to concern for apnea, previous studies have shown that infants who require transfer to a tertiary center on a PGE_1_ infusion should be intubated prior to transfer [13]. However, no transfer guidelines have been established to date, and recent data have also shown that the risks of intubation prior to transport of stable infants on low-dose PGE_1_ must be considered carefully against possible benefits [14, 15].

## 3. Conclusions

Coarctation of the aorta is well understood and characterized, yet it continues to be the most commonly missed congenital heart disease. Presentation typically occurs during the first two weeks of life, when the lesion is at its most critical state and when mortality rates are at their highest. With the majority of CoA being ductal-dependent lesions, closure of the ductus during the first 2 weeks of life can lead to rapid clinical compromise. For this reason, pediatric emergency room physicians should initiate PGE_1_ in newborns with suspected sepsis and isolated tachypnea until CoA and CHD are ruled out.
